# A Data Quality Control Program for Computer-Assisted Personal Interviews

**DOI:** 10.1155/2012/303816

**Published:** 2012-12-10

**Authors:** Janet E. Squires, Alison M. Hutchinson, Anne-Marie Bostrom, Kelly Deis, Peter G. Norton, Greta G. Cummings, Carole A. Estabrooks

**Affiliations:** ^1^Clinical Epidemiology Program, Ottawa Hospital Research Institute, Ottawa, ON, Canada K1H 8L6; ^2^School of Nursing, University of Ottawa, Ottawa, ON, Canada K1H 8M5; ^3^Cabrini-Deakin Centre for Nursing Research, School of Nursing and Midwifery, Deakin University and Cabrini Health, Melbourne, VIC 3KM, Australia; ^4^Division of Nursing, Department of Neurobiology, Care Sciences and Society, Karolinska Institute, 14183 Huddinge, Sweden; ^5^Department of Geriatric Medicine, Danderyd Hospital, 18287 Danderyd, Sweden; ^6^Faculty of Nursing, University of Alberta, Edmonton, AB, Canada T6G 1C9; ^7^Department of Family Medicine, University of Calgary, Calgary, AB, Canada T2M 0H5

## Abstract

Researchers strive to optimize data quality in order to ensure that study findings are valid and reliable. In this paper, we describe a data quality control program designed to maximize quality of survey data collected using computer-assisted personal interviews. The quality control program comprised three phases: (1) software development, (2) an interviewer quality control protocol, and (3) a data cleaning and processing protocol. To illustrate the value of the program, we assess its use in the Translating Research in Elder Care Study. We utilize data collected annually for two years from computer-assisted personal interviews with 3004 healthcare aides. Data quality was assessed using both survey and process data. Missing data and data errors were minimal. Mean and median values and standard deviations were within acceptable limits. Process data indicated that in only 3.4% and 4.0% of cases was the interviewer unable to conduct interviews in accordance with the details of the program. Interviewers' perceptions of interview quality also significantly improved between Years 1 and 2. While this data quality control program was demanding in terms of time and resources, we found that the benefits clearly outweighed the effort required to achieve high-quality data.

## 1. Background

Good data quality is fundamental to survey research; poor data quality can provide misleading results and seriously invalidate study findings. Hence, researchers will often expend considerable effort on quality control procedures. Factors contributing to data quality are numerous, complex, and multidimensional [1]. Sources of error include coverage, nonresponse, sampling, respondent, instrument, and mode of delivery [2]. In the case of face-to-face interview data collection methods, including telephone and computer-assisted personal interviews, the interviewer is also an important part of the process and can be a further source of error.

Two common approaches to survey data quality are the total quality management (TQM) approach and the total survey error (TSE) approach. The TQM approach to data quality focuses on the process of survey production and is based on the assumption that the quality of all elements of the production process contributes to quality of the final dataset [1]. According to this approach, data quality is a function of not only accuracy but also the relevance, comparability, coherence, timeliness, and completeness of the data. Evaluation of quality in the case of TQM addresses process and outcomes. The TSE approach, on the other hand, describes quality in terms of accuracy and defines data quality “as the relative absence of systematic variable errors” [1, page 66]. Finally, Loosveldt and colleagues emphasize the value of a pragmatic approach to data quality, which focuses on evaluation of the survey process and outcomes as well as on the interviewer tasks, thereby integrating the TQM and TSE approaches [1]. 

While there is some literature describing quality control and assurance procedures for clinical trials (e.g., Martin and colleagues [3]), this literature is scarce and does not fully apply to survey research. The purpose of this paper is therefore to describe a data quality control program (with elements of TQM and TSE approaches) that was developed to maximize the quality of survey data collected using computer-assisted personal interviews (CAPIs). CAPI involves interviewers reading survey questions aloud to participants and entering responses directly into a survey application using a computer. We illustrate our data quality control program by describing the program and evaluating its usefulness with survey and process data collected in the *Translating Research in Elder Care* (TREC) Study [4, 5]. In TREC, all healthcare aides in 36 nursing homes (in Western Canada) who met the study inclusion criteria were invited to complete the TREC survey (a suite of self-report instruments) annually for two years (June 2008–July 2010). Data collection occurred in quarters with each nursing home having data collected in the same quarter each year. Trained TREC research staff administered the survey to healthcare aides using CAPI. Where it was not possible to use CAPI (e.g., rare situations in which the interviewer could not open the computer software application), a paper survey was completed and data were later entered into the virtual system. Further details on the TREC data collection procedures are reported elsewhere [4].

## 2. Methods

### 2.1. The Data Quality Control Program

**Figure 1:**
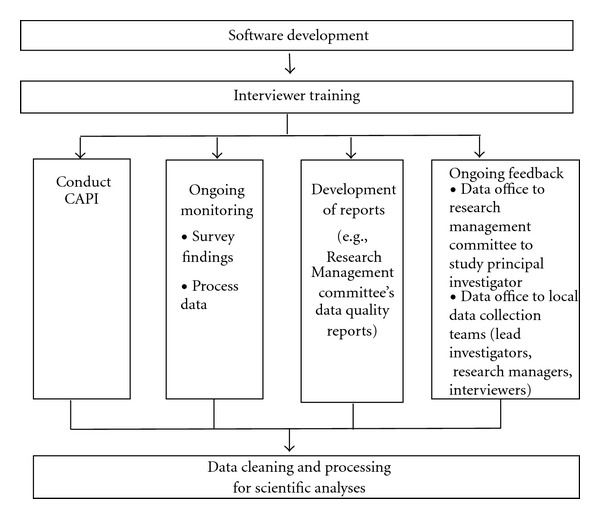
Data quality control program.

The data quality control program comprised three phases: (1) software development, (2) an interviewer quality control protocol, and (3) a data cleaning and processing protocol (Figure 1).

#### 2.1.1. Phase 1: Software Development

We contracted the services of a Canadian-based software developer with experience in the development of online surveys and an understanding of the health sector (https://nooro.com/). Our requirements were complex; providing respondent and interviewer access to the survey using a variety of computer systems located within diverse environments across geographically distributed locations and all systems requiring capacity to securely upload individually gathered data to the master dataset located on a remote server. This is typically where Internet accessible surveys would be well suited; however Internet access in our settings was inconsistent and in some communities nonexistent. Without reliable Internet access, our best solution was to purchase several laptop computers (for use by interviewers undertaking data collection concurrently in each setting) and have the survey software installed on each laptop. This allowed for offline survey completion and temporary storage, with subsequent upload of data using a secure file transfer service when internet connectivity was available. Each interviewer was allocated a unique identifier, and each data file had a unique file naming convention.

Important considerations for the software development process included maintenance of confidentiality across the sites and provinces, minimization of error, and expedience in conducting the survey. To help meet these requirements, one feature of the survey software involved predetermined options for certain fields. This feature was linked to the interviewer's unique identifier so that each interviewer would only see options that were relevant to their jurisdiction (e.g., when they selected *facility name*, the common names of the units *only* within that facility would appear). We also recognized that cultural, environmental, and personal experiences could result in differing understandings of terms and phrases used in the survey. To aid understanding, promote standardization, and minimize the influence of interviewer bias, tips and prompts were built into the survey in select places. Interviewers were instructed to use these prompts only if a term or phrase was not clear to the respondent. If a prompt was not available, interviewers were instructed not to say anything additional, to ensure standardization of delivery of the survey.

A risk of CAPI is that the interviewer might inadvertently omit questions, resulting in missing data. To help overcome this, a check and balance system was implemented, whereby at the end of the survey a screen would appear advising the interviewer of the number of questions that remained unanswered and their location within the survey. The interviewer then had the option of returning to the questions concerned to confirm whether they were missed, as opposed to refused by the respondent, and if so, to obtain a response.

Another important consideration in the software development process was the data upload procedure. Development, refinement, and testing of this process took into account the steps required to connect to the server, how soon after data collection the data had to be uploaded, and the type of confirmation that was generated to signal a successful upload. Additionally, system checks were put in place to ensure the same data could be uploaded only once. Testing of the survey and the upload process to ensure each was fully operational and behaving as expected involved internal and external review phases. The internal phase involved a cycle of development, review and testing, and modification, followed by further review and testing. This process took into account the survey content, visual appeal of the survey, and ease of navigation. The former involved checking the question order, completeness of questions, spelling, grammar, and punctuation. The latter involved consideration of overall appearance of the survey, the colors, the format and layout of responses, the number of questions per page, how the questions were separated (different colors and width of lines), the ability to advance or move back in the survey, and the ability to change responses. The external phase involved testing of the software by healthcare aides and evaluation of the overall appearance, functionality, and ease of navigation within the survey.

The final stage of the software development addressed capacity for “real time” monitoring to ensure high-quality data. The software developer provided a private site, accessible only by authorized TREC administration staff, which allowed for generation of live, standardized reports of the number of surveys completed by setting (e.g., province, site). An important feature of the CAPI system is the capacity to generate paradata (data relating to the process of data collection). This data included the number of attempts interviewers had at completing an individual interview, the length of time each interview took, and the time of day the interviews were conducted. Such data enabled tracking of interviewer performance, which is important to achieving high-quality data.

#### 2.1.2. Phase 2: Interviewer Quality Control Protocol

Each of the three provinces participating in the TREC study established a local data team, which was responsible for healthcare aide recruitment and data collection. Each team was led by a site investigator and included a research manager and one or more research assistants and/or professional interviewers.

The data teams participated in intensive interviewer training to ensure standardized technique and the collection of high-quality data. To facilitate this process, an interviewer (procedure) manual and an interviewer quality control protocol were developed and implemented as components of the data quality control program. The interviewer manual contained technical information on the TREC study, the survey, the step-by-step process of conducting a CAPI interview, and an overview of the CAPI software and the processes by which the data were to be handled. The interviewer quality control protocol (Supplementary File 1; see supplementary materials available online at doi:10.1155/2012/303816) was central to the quality control program and contained three core components: (1) characteristics of a successful interviewer, (2) training, and (3) tracking and monitoring processes.

**(1) Characteristics of a Successful Interviewer**

Four broad categories of characteristics of a successful interviewer were identified based on a review of existing literature and our experience with conducting face-to-face structured interviews. The four categories were (1) physical attributes, (2) personal characteristics, (3) technical skills, and (4) compliance with interview procedures. Physical attributes included open posture, consistent eye contact (with interviewee), and comfort with conducting the interview. Personal characteristics included a personable demeanor, engaging with the interviewee, appropriate speed of talking and clear and audible speech, appropriate (professional) dress and hygiene, and ability to problem-solve (e.g., technological problems) during interviews. Technical skills included ability to log on to the computer, ability to open and launch the virtual server CAPI software, ability to navigate through the survey, acceptable typing speed, ability to conduct the interview while entering responses with minimal delays, and ability to connect to the virtual server to allow data synchronization and upload following the interview.

**(2) Interviewer Training**

The interviewer training consisted of two core elements: (1) a field school or orientation session, depending on date of hire of the interviewer, and (2) practice interviews. Explicit steps were taken to standardize interviewer techniques in order to maximize consistency between interviewers. Initial training included attendance at a two-day “CAPI Field School.” All existing and newly hired staff (research managers and research assistants) who could potentially be assigned to conduct CAPI interviews for the study were required to attend the field school. The study principal investigator, provincial investigators, administrative staff, and research trainees also attended the field school, which took place one month prior to initiation of data collection. The intent of the field school was threefold to ensure interviewers (1) had a shared understanding of the study, (2) understood how to use the CAPI software, and most importantly (3) received the same (standardized) training with respect to *how* to conduct CAPI. Interviewers hired after the field school were required to attend an orientation session in their province, hosted by the provincial research manager, which incorporated the important elements of the field school.

The field school was developed to be informative, interactive, fun, and a team building process. Sessions focused on (1) getting to know the research team, (2) providing information on the TREC study and survey (including an item-by-item review of the survey to ensure all potential interviewers understood the items in the same way) and the CAPI and software process, and (3) practice interviews. Prior to practicing their interviewing skills, field school participants observed “good and easy” and “bad and difficult” interviews as role-played by TREC administrative staff and research trainees. A “bad and difficult” interview was role-played first. Participants were then asked to provide feedback in terms of what could have been done differently. The same role players then conducted a “good” interview to highlight the way in which data could be collected more efficiently and effectively. Following role-playing, participants were assigned to small groups where they were asked to rotate through the roles of interviewer, interviewee, and observer. A senior investigator circulated throughout the groups observing, giving feedback, and answering questions. To conclude the initial (field school) training, all team members were invited to share their experiences with the group.

Following attendance at the field school or orientation session and prior to conducting formal data collection, each interviewer was required to complete a minimum of five practice interviews in which they demonstrated an acceptable level of competence and the characteristics of a good interviewer. Three interviews were done with people other than other interviewers (e.g., investigators) and two were with other interviewers. A minimum of two of these interviews was required to be observed by the provincial research manager who provided feedback on the interviewer's performance using standard forms: an *interviewer checklist* (which outlines the characteristics of a good interviewer) and an *interviewer monitor form* (which lists interviewer techniques, delivery, and data entry skills) (both in Supplementary File 1). In some cases, the research manager also attended the first few “real” interviews to ensure compliance with the interviewer quality control protocol.

**(3) Monitoring and Feedback**

Information on the quality of the survey data collected in the CAPI interviews and the process of conducting the interviews was monitored throughout the data collection period. Monitored information included survey findings (e.g., missing data, skewness) and process-related data (e.g., travel time, time on site, number of interviews completed/in progress/refused) collected using standardized forms, which were submitted to and verified by the central office for the TREC study. In the event of discrepancies or errors with the process data, the data manager for the study would contact the research manager for the indicated province for resolution. Once verified, the information was entered (and double checked for accuracy) into a statistical database where it was analyzed and used to generate quality reports. Security and confidentiality policies were enforced for all reports (e.g., forms had to be sent by bonded courier; courier packages had to be received by an identified person in central office and were documented and stored in a locked cabinet). Also as a part of the quality control program, following each interview (once the respondent (healthcare aide) left the room), interviewers were asked to complete a series of questions (the *interviewer checklist*, Supplementary File 1) on the interview process. This also allowed for a better understanding of the circumstances in which each survey was completed. These data were analyzed regularly (quarterly) to further assess quality of the interviews and compliance with the quality control interviewer protocol. This information was fed back to the interviewers when necessary.

**Figure 2:**
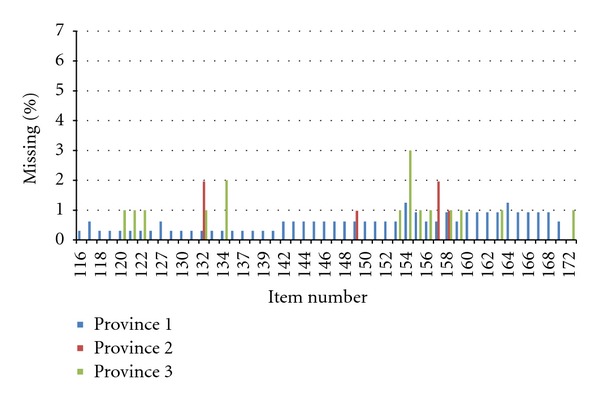
Missing values by item for all provinces (July 16th, 2009–Nov 23rd, 2009).

**Figure 3:**
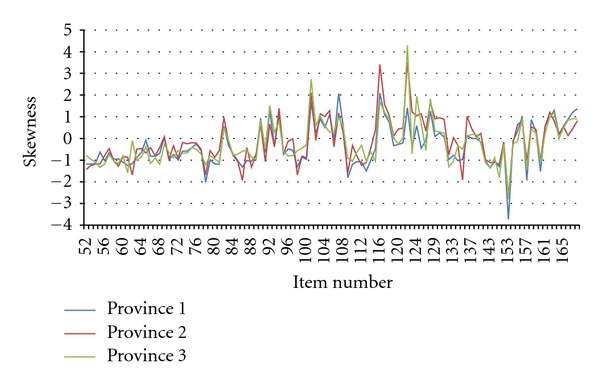
Skewness by item for all provinces (July 16th, 2009–November 23rd, 2009). Comment presented in the report: The skewness graph indicates if the answers to any item are skewed or not. Negative is left skewed (tend to have small values) and positive is right skewed (tend to have large values). In the skewness it shows that there is no significant difference among all three provinces.

Regular feedback on the quality of the data to the TREC Research Management Committee and the local (provincial) data collection teams was a critical component of the interviewer quality control protocol. The Research Management Committee was composed of the study's principal investigator, senior investigators, and decision makers. The committee met quarterly. A CAPI data quality report was prepared for and reviewed at each Research Management Committee Meeting throughout data collection. This report included, for example, for each interviewer the number of interviews completed, missing data by survey item, item skewness and kurtosis, and instances where survey responses were significantly different for one interviewer compared to other interviewers within a facility and/or province.  Table 1 and Figures 2 and 3 comprise a sample table and graphs from a data quality report.

Ongoing feedback was also provided to the local data collection teams. Interviewers were given feedback starting the first day after data collection and regularly thereafter. Details of the feedback provided at each time interval (i.e., weekly, quarterly, yearly) are summarized in the quality control interviewer protocol (Supplementary File 1). In addition to this feedback, data-related issues such as missing data and survey item responses that differed significantly from other interviewers were also fed back to the provincial lead investigator in each province who discussed the issue with their research manager and interviewers.

#### 2.1.3. Phase 3: Data Cleaning and Processing Protocol

The third and final phase of our quality control program was a data cleaning and processing protocol (Supplementary File 2). The protocol consisted of six steps and was implemented quarterly by a data analyst for the study: (1) systematic data entry, (2) data cleaning, (3) prederivation processing, (4) derivation of scale scores, (5) descriptive assessment of derived scores, and (6) assessment of missing data. Throughout this process, a four-part report was produced for the study lead investigators: steps 1-2 (report A), step 3 (report B), steps 4-5 (report C), and step 6 (report D). Each report was reviewed and approved by the study principal investigator before the data analyst proceeded to the next phase of cleaning and processing. 

## 3. Results

### 3.1. Survey Data

#### 3.1.1. Missing Data

 Data were collected from 3004 healthcare aides (1494 and 1510 in Years 1 and 2, resp.). Missing data was minimal with 99% of healthcare aides having 5% or less missing data (Table 2). Individually, all cases with >10% missing data (i.e., missing on at least 19 of the 192 survey items) were explored to inform a decision regarding retention. A total of 12 cases (0.4% of the sample) had >10% missing data; 8 of these cases were deleted because data were missing for several core domains that were essential to the testing of the study's main hypotheses. One additional case was also deleted from the data because the participant did not meet the eligibility criteria. This resulted in a final sample size of 2,995 (99.7% of all healthcare aides interviewed). 

#### 3.1.2. Systematic Data Errors

Systematic data errors (i.e., errors that tend to shift all measurements in a systematic way [2, 6]) were corrected within the data as they were discovered. Such errors were minimal (*n* = 6) and most (*n* = 5 of 6) related to miscoding of missing and/or not applicable responses. Outside these coding errors, only one systematic error was detected. The responses for one set of six items were all 1's across all data. After consultation with our software developer, it was discovered that the original codes for the item set were imported incorrectly into the software; a new code was written by the software developer to correct the error.

#### 3.1.3. Random Data Errors

We also assessed for random data entry errors where paper surveys were used. Random error, also known as variable or chance error, is caused by chance factors that confound measurement [2]. A total of 70 paper surveys were completed (43 in Year 1 and 27 in Year 2). Annual “random” data entry error rates were low: 13% and 1.6% for Years 1 and 2, respectively. The annual random error rate was calculated using the following formula: number of errors/[(192 items)(X surveys)]. Since we checked all entries on all paper surveys, we also corrected all random errors.

#### 3.1.4. Distributions

As a part of our ongoing monitoring of the survey data, we also examined each item (and scale scores) quarterly for mean and median values, standard deviation, skewness, and kurtosis. All mean and median values and standard deviations were within acceptable limits. Skewness and kurtosis was minimal with the majority of items displaying an approximate normal distribution. 

### 3.2. Process (Interviewer) Data

Process-related data collected from the interviewers included, for example, whether or not a paper-based survey was used. These data indicated that the proportion of paper-based surveys used was small and fell over time (from 2.9% in Year 1 to 1.8% in Year 2; chi-square, *P* = 0.040): an indication that the procedure for enabling software updates overnight (to prevent computer start-up delays during the daytime) was functioning well and the interviewers were confident in using the software. Data were also collected on whether or not the interviewers were able to set up the interview according to protocol. Overall, in only 3.4% (Year 1) and 4% (Year 2) of cases was the interviewer unable to set up in accordance with the protocol. On most occasions, the interview was conducted in a private location as per protocol (72% in Year 1 and 78% in Year 2). Frequently the location was also visible to other staff (65% in Year 1 and 75% in Year 2) and close to resident care (68% in Year 1 and 86% in Year 2). Interruptions during data collection, which could potentially threaten the quality of the data, were also monitored. The majority of the interviews were conducted without interruption (76% in Year 1 and 84% in Year 2). Another possible threat to data quality was pauses (where the interview had to be stopped and restarted). The majority of interviews proceeded that required a pause (91% in Year 1 and 95% in Year 2). Interviewers were also asked to rate the overall quality of the interview from 1 (terrible) to 5 (wonderful). Their perceptions of overall quality improved between Year 1 (mean 3.84) 2 (mean 4.11); this improvement was statistically significant (chi square, *P* < 0.001) which could reflect improved competence and confidence from training and feedback provided in the data quality control program and as they gained experience in conducting the interviews. 

We also examined survey data in relation to the individual who conducted the interview. This was part of the quarterly quality reports. In particular, we assessed the data (by interviewer) for missing data and skewness to determine if we had any “interviewer problems.” Overall, few issues were noted. Some instances that were detected are as follows. In one instance, we discovered skewed healthcare aide responses for a particular item set for the majority of the interviews conducted by one interviewer in one quarter. The information was feed backed to the local team and the provincial research manager then observed the interviewer conducting their next set of interviews. This revealed that the interviewer was delivering (reading) the questions too quickly, resulting in the healthcare aides not having sufficient time to answer the questions with accuracy. The interviewer was given feedback accordingly and the skewness of his or her data was monitored in future reports. No further problems were observed, illustrating the importance of our quality assessment and providing ongoing feedback to the interviewers. In another instance, high levels of missing data for specific variables, across interviewers and provinces, were detected. Investigation revealed that this issue was related to a security update performed by the software provider, which had affected specific data fields during the survey upload process. This problem was rectified and the correct data were restored. In another example, a quality report highlighted the existence of relatively high rates of missing data on the interviewer checklist across several interviewers. The checklist data were very important for assessment of the overall quality of the interviews. In response to identification of this discrepancy, the research managers stressed to the interviewers the importance of completing the checklist. Subsequent reports found almost no missing data for the interview checklists. 

## 4. Discussion 

Our efforts to adopt the CAPI method, to provide interviewer training, and to implement a rigorous quality control program were motivated primarily by a desire to promote efficiency in the collection and processing of the survey data, maximizing the quality of the data, facilitating data processing, and saving time. The upfront cost (in terms developing the software and training the interviewers) and ongoing investment (in terms of staff and investigator person hours required to develop the quality reports and clean/process the data) was not insignificant. For instance, approximately 500 hours of paid staff time (combined data analyst, data manager, and data research assistant) were required to compile the quarterly quality reports and clean and process one year of CAPI data. However, despite this investment of time and resources, our results demonstrate that the CAPI method combined with our data quality control program was successful overall, which for us made this investment worthwhile. Missing data was minimal with only 8 cases (out of a possible 3004) requiring deletion due to excessive (>10%) missing data; we believe this result can be attributed to a combination of the survey method, the software design, interviewer skills, and regular monitoring. Our ongoing monitoring process (e.g., quality reports and data cleaning and processing reports) further enhanced our ability to produce high-quality data for the study. The generation of the quality reports was unique to the TREC study. These reports were highly beneficial in highlighting aspects of data quality that required further investigation such as high levels of missing data for specific variables and/or across interviewers. In several cases, had a quarterly quality report not been generated, it is unlikely that certain issues would have been detected and thus corrected.

As recommended by Loosveldt and colleagues [1], we adopted a pragmatic approach to the promotion of data quality, integrating the TQM and TSE approaches to focus on evaluation of the survey process, outcomes, adnd interviewer tasks. Interviewer skills cannot be assumed to be of high-quality, and variability in interviewer proficiency presents a significant risk to data quality. While training can address interviewer skills, variability between interviewers cannot be overcome by training alone [7–11]. Thus, use of a standardized approach to data quality control that addressed process as well as outcome measures provided us with ongoing monitoring of the quality of our survey data and enabled us to intervene when deviations or discrepancies were identified. Additionally, the use of CAPI enabled further standardization and control of the interview process by allowing for automated skip patterns and embedded prompts. Additionally, prompts at the completion of the survey to alert the interviewer to any unanswered questions helped to promote completeness of the data. In combination, we found that these strategies maximized the quality of the survey data we collected.

## 5. Challenges Encountered

Despite the benefit of acquiring high-quality data, our quality control program was not without its challenges. Internet access, interviewer training, maintaining security of the data, and the monitoring and tracking of the data presented a range of challenges for the investigatory team to overcome. These challenges were not insurmountable, but they did require a substantial lead-time to address them adequately prior to the commencement of data collection. For example, the decision to adopt the CAPI approach to data collection presented challenges with respect to Internet access. Because reliable Internet connections could not be guaranteed, the software had to be developed to enable delayed transmission of collected data. Data security was of a high priority for data stored on laptop computers until such time as the interviewer could access the Internet to upload their data. As a result, strict procedures and protocols were required to promote consistency in handling of the computer equipment and transfer of the data in order to reduce the risk of a security breach or loss of data. Further, computers purchased within each of the provinces had different operating systems and there were varying security arrangements across the sites. This resulted in the software developer being responsible for specific installations and instructions for each software user to ensure all computers functioned correctly.

While interviewer training was labor intensive, it was a vital element of the quality control process. This training involved not only development of interpersonal and interviewing skills but also skills in use of the equipment, navigation of the survey, and transmission of the data following the interview. An unplanned delay for two of the three participating provinces between the field school and the commencement of the interview data collection phase enabled interviewers to practice, but was a threat to skill retention. Field school was only offered at the startup of the interview data collection phase, and ongoing training of interviewers who were dispersed over three provinces had to be undertaken at the provincial level. This requirement had the potential for some variation in training, even though a standard training manual was provided. Additionally, as new interviewers were employed, training had to be provided on an ad hoc basis, as required. A further challenge was that interviewers only had access to the software developer during normal business hours. Therefore, if difficulties were encountered in the field, outside business hours, interviewers often could not achieve a resolution in a timely manner. This, however, was rare.

## 6. Conclusion

The quality of survey data is of methodological importance and can be addressed using a comprehensive, standardized quality control and improvement process. Our findings indicate that the data quality control program developed in the TREC study can have a positive influence on data quality. While there are many challenges associated with achieving high survey data quality, the benefits outweigh the effort required to achieve high-quality data.

## Supplementary Material

Supplementary File 1: The interviewer quality control protocol describes three core components of interview quality control: (1) characteristics of a successful interviewer, (2) training, and (3) tracking and monitoring processes.
Supplementary File 2: The data cleaning and processing protocol describes a six step process: (1) systematic data entry, (2) data cleaning, (3) prederivation processing, (4) derivation of scale scores, (5) descriptive assessment of derived scores, and (6) assessment of missing data.

## Figures and Tables

**Table 1:** Number of interviews for data collector by province and nursing home, partial wave 2 (July 16th, 2009–November 23th, 2009).

Data collector	Province																		Total
	1									2					3				
	Facility									Facility					Facility				
	S3	A2	T5	E5	B9	H3	G7	D2	Total	R7	W9	F3	K4	Total	Z8	T9	Q8	Total	
1	11		27					36	74										74
15					8	4	24		36										36
16					28	16	41		85										85
29	2								2										2
30	12	10	20	2				26	70										70
35		9	37	8					54										54

26										9	4	13		26					26
34										25	16	22	13	76					76

13															6		7	13	13
18															28	6	17	51	51
27															21	9	6	36	36

Total	25	19	84	10	36	20	65	62	321	34	20	35	13	102	55	15	30	100	523

**Table 2:** Missing data over the two years of the TREC Project 1.

Missing rate	Year 1		Year 2		Total (Year 1 + Year 2)		
	Frequency	Percent	Frequency	Percent	Frequency	Percent	Cumulative percent
No missing	0	0	1135	75.2	1135	37.8	37.9
0%~1%	0	0	222	14.7	222	7.4	45.3
1%~2%	0	0	95	6.3	95	3.2	48.5
2%~3%	1010	67.6	33	2.2	1043	34.7	83.3
3%~4%	381	25.5	8	0.5	389	13.0	96.3
4%~5%	70	4.7	8	0.5	78	2.6	98.9
5%~10%	26	1.7	3	0.2	29	1.0	99.9
>10%	6	0.4	6	0.4	12	0.4	100.0

Total	1493	100.0	1510	100.0	3003	100.0	
