# Supplementary material for: A Data Quality Control Program for Computer-Assisted Personal Interviews
Source: Nurs Res Pract. 2012 Dec 10;2012:303816. doi: 10.1155/2012/303816 (PMC3529418; doi:10.1155/2012/303816)
Supplement: Supplementary file 1 — The interviewer quality control protocol describes three core components of interview quality control: (1) characteristics of a successful interviewer, (2) training, and (3) tracking and monitoring processes. Supplementary File 2: The data cleaning and processing protocol describes a six step process: (1) systematic data entry, (2) data cleaning, (3) prederivation processing, (4) derivation of scale scores, (5) descriptive assessment of derived scores, and (6) assessment of missing data. [file 303816.f1.doc]

# Supplementary File 1

# Interviewer Quality Control Protocol

**TREC Project 1**

# Prepared by:

# The *Translating Research in Elder Care (TREC)* Program

# Quality Matters!

# TREC Protocol: Interviewer Quality Control

## Preamble

Researchers have an obligation to be fiscally responsible and therefore, quality control processes should be implemented at multiple levels to ensure data collected is of good quality and will provide information to help answer the research question(s). The worst-case scenario is to be at the end of your funding period and find your data cannot be analyzed and funds have been irresponsibly spent. Quality control is an important issue for any type of investigation, whether single – or multi-centred.

Quality control is important for a number of reasons but initially it sends a message to interviewers that their work is valued and followed, and that the researcher attaches great importance to the way in which the interviewers carry out their tasks. It is intended to motivate, not punish, the interviewer.

## Current Situation

In TREC (Project 1), training for interviewers has been determined as a primary activity to ensure standardized interviewer technique (that is, between interviewers and across provinces) ensuring that interviews of comparable respondents are taking place in a consistent manner. Targeted funding has been set-aside in 2008/09 for the initial training and protocol development.

The initial group of TREC interviewers comes from diverse backgrounds with none to several years experience conducting structured phone or face-to-face interviews and/or qualitative interviews. None have done structured face-to-face interviews using computer recording (CAPI: computer assisted personal interviews) in the past.

In 2008/09, we expect to interview approximately 1440 aides (40 x 36 facilities) across the three Prairie Provinces. In Manitoba (MB) and Saskatchewan (SK), the respective research managers will assist with interviewing. In Alberta (AB), due to the number and size of sites, the research manager will focus largely on overseeing scheduling, **quality control**, and troubleshooting. We are currently funding 2 P/T interviewers in MB; 1 F/T in SK and the equivalent of 3.5 F/T in AB. For a number of reasons AB chose to use a mixture of research assistants hired by TREC and contracted interviewers who are paid by the hour. A combination of staff and contractors will conduct interviews in Capital Health Region and East Central Health Region while only contracted individuals will conduct interviews in Calgary Health Region sites.

The broad aims of training are to:

1) Reduce measurement errors that may take place when registering responses;

2) Minimize interviewer effects, and

3) Assist the interviewer and ultimately the respondents to complete his/her task in a timely and adequate fashion.

Areas of training focus are:

- Rules for conducting interviews
- The method by which one gives precise, systematic instructions and positive/negative feedback
- Interviewer reaction to inadequate respondent behaviour

The training of interviewers, the way interviewers convert their training into practice, and the feedback received by interviewers in the course of conducting interviews are all-important in process evaluation. The following describes the various phases and the tools to assist in providing feedback.

## Characteristics of a Successful Interviewer:

The TREC team has identified characteristics of a successful interviewer that reflect physical attributes, personal characteristics, technical skills, and compliance with TREC policies. The following outlines these areas in more detail:

- Physical attributes:
- Open posture
- Regular eye contact with interviewee
- Comfort with response card format
- Personal characteristics
- Interviewers have interviewed a comparable group of respondents resulting in comparable average interview duration. The goal is ~20 minutes for the survey itself (not including consent process, which can be done at a separate time on the same day).
- Personable, engaging, professional
- Appropriate speed of talking; no stumbling; pauses
- Appropriate dress
- Good hygiene
- Clear and audible
- Confident with problem-solving during interviews
- Technical skills:
- Able to log on to the computer, knowing the IDs/passwords for that machine
- Able to open and launch the virtual server software developed specifically for this project
- Able to read and accurately enter the unique identifier
- Comfortable with using a mouse
- Acceptable typing speed
- Able to conduct the interview while entering responses with minimal delays
- Understands the software sufficiently that there are no surprises as data is entered (e.g., “I wonder why that icon just showed up?”)
- Able to open the Internet and connect to the live server to allow data synchronization
- Compliance with TREC policies for:
- Obtaining consent
- Confidentiality
- Conducting interviews (e.g., no personal information; no comments to responses)
- Completion of required tracking forms
- Uploading data
- Secure and safe handling/storage of equipment/documents (e.g., laptops)

## Initial Training

Because each province in the study hired personnel with a variety of skills, initial training was mandatory to ensure quality control at all points in the interview procedure. TREC Central believed it was important that training take place at one center over two days. All staff conducting interviews (plus key lead investigators and administrative staff) met in Winnipeg May 12 and 13, 2008. This exercise was a challenge because the software was not fully functional and contingencies had to be put in place to enable use of the computers for training purposes. In preparation for the event, a procedure manual was developed providing orientation to the project overall and long term care, in particular.

The intent of the training was to ensure everyone had a shared understanding the project and the software and also to solicit input in terms of what worked or not as they practiced their interviewing techniques. Interviewers observed a ‘bad’ interview and then provided feedback in terms of what could have been done differently. The same players then conducted a ‘good’ interview to highlight the way in which data could be collected more efficiently and effectively. Over the course of the training, interviewers were assigned to small groups and rotated through the roles of interviewer, interviewee and observer. After a few rotations interviewers conducted two interviews on their own, practicing the skills taught throughout the training. To conclude the initial training, team members were invited to share their experiences and debrief.

All feedback was recorded and immediately upon return from the ‘field school’, discussed, and agreements made about which recommendations to implement, how and in what timelines. Prior to the TREC annual meeting on June 5 & 6, 2008, the health care aide version of the TREC Survey had been updated and the majority of amendments made. The final stage was to create the virtual machine software and install it on all TREC laptops in preparation for data collection.

The following describes additional training required before data collection can begin. Any new staff member must follow all these steps before they enter the field.

## Before data collection begins

- Research Managers will **orient** interviewers to the:
  - Recruitment process
  - Web survey formats for regulated and non-regulated staff
  - Web survey packages (and processes specific to HCAs)
  - Virtual Server: what it is, how to access, close and upload data
  - Consent process
  - Response cards (and when/how to use)
  - Required tracking forms
  - The standard software used for data collection
- Interviewers must **read** the following documents:
  - Protocol 1(*only if TREC staff; not required of contracted interviewers*)
  - Interviewer manual including:
    - Orientation to Long Term Care
    - TREC policies
    - Question by Question guide
    - Software training
  - HCA survey (print copy of scripted version)
- Interviewers must do a minimum of **5 training interviews and demonstrate an acceptable level of competence**
  - 3 with people other than other interviewers (e.g., staff, investigators, support staff, students and/or family members)
  - 2 with other interviewers (due to staff numbers, it may be with each other!)
  - A minimum of 2 of these interviews must be observed by the Research Manager; 1 in each of the above categories to provide different perspectives.
  - If the Research Manager is also conducting interviews, they must have 2 interviews observed by the provincial lead investigator
  - Where possible, record interviews and listen to them afterwards as part of a feedback mechanism
- Interviewers must **demonstrate the previously described characteristics of a successful interviewer**.
- If an interviewer **does not** demonstrate the **characteristics of a successful interviewer**, further training will be required before entry to the field. The number of additional training interviews required will be set at the discretion of the Research Manager:
  - If the Research Manager determines that select, limited aspects of the protocol need to be reinforced, one additional training interview must be conducted. The RM will check up closely after the first day of data collection to ensure areas of concern were addressed.
  - If the Research Manager determines that several additional training interviews are warranted, then the Research Manager is required to attend the first 2 ‘real’ interviews to ensure compliance with protocol; in the case of the Research Manager being the interviewer, the provincial lead investigator is required to attend the first 2 ‘real’ interviews.
  - If the Research Manager determines that repeated training interviews are required, then consideration should be given to replacing this person.

## After 1^st^ day of Data Collection

- After the first ‘upload’ of data, each interviewer will meet (in person or over the phone) with their respective Research Manager to review the process and answer any questions. The Research Manager will ask questions such as:
  - Did you have any problems with the location and/or finding your respondents?
    - Did you have a private room/space?
    - Were you interrupted during the interview(s) either by staff, residents or volunteers?
  - How long did your interviews take?
    - Did you encounter any difficult questions or situations?
    - How did you handle that (…a particular question or situation)?
    - What did you say?
    - Why did you do that?
  - What will you do if something always comes up around a question but the manual isn’t clear on what to do? Where will you go for the answer?
  - Did you have any problems with the software?
    - Could you connect to the survey and perform the survey using the software?
    - Did prompts come up when you clicked on the “i” symbol? When did you use them?
    - Were the ‘mouse overs’ helpful?
    - Were you able to upload your survey results to the central server (and how would you know)? Where did you do the upload (in the office, at home, at a café, etc)?

**NOTE:** Research Managers meet weekly by phone and will discuss any common themes and issues that emerge across provinces and sites. If questions arise between meetings that the research manager cannot answer, an email should be sent to the Administrative Director stating the question and asking for a response. All Research Managers will be copied on the response and the manual updated accordingly, to include advice and protocols to address the question/issue raised.

## Weekly

- Interviewers must meet with the Research Manager (RM) to debrief their experiences….not just with the survey and the tool but also the experience of going into LTC settings. Because schedules need to be distributed on a regular basis, it might make sense to do this at the same time.
- Research Managers will discuss survey completion times with each interviewer, assessing which interviewers deviate significantly from the average interview duration. Significant deviation may require intervention. Items to consider are:
  - The way in which the questions are asked
  - Appropriate reference being made to response cards (where required)
  - The way in which instructions and clarifications are given to the respondent
  - The speed at which the interview was conducted.
  - Degree to which the interviewer has applied the content of their training when conducting interviews.
- Interviewers are encouraged to contact their Research Manager if they have a particularly challenging experience within an interview or site.

## Quarterly

- Research Managers will observe, at random, the interviewer during data collection. A minimum of one check/interviewer/quarter is **required**. More frequent observations will occur if the Research Manager has any indication of deviation from the standard interview protocol or problems during interviews. The Research Manager will observe the interview from beginning to end. The following items should be considered:
  - The interviewer should inform and gain the respondent’s consent for the supervisor to sit in during the interview. It would be helpful to say something like “*My supervisor has to observe me doing an interview as part of our quality control. Is it OK if he/she sits in today?”* If they do not agree, then the Research Manager should leave the room.
  - The Research Manager should maintain an observation ‘status’ and sit between the interviewer and respondent (that is not behind or to the side but rather as the 3^rd^ point of a triangle) so they can observe both the interviewer and interviewee. The Research Manager should not interfere with the interview at all or interrupt the connection between the other two parties.
  - The **TREC Survey Interviewer Monitor Form (Internal form 2)** can be used to assist the Research Manager in providing feedback. The main reason for doing quality checks is to determine that the protocol is being followed.
  - If there is a serious breach in protocol debriefing must occur immediately following completion of the interview; otherwise debriefing can occur at a normal break in the day. If issues are minor (e.g., talk slower/faster; let them see the screen for a few seconds more, etc) then just inform the interviewer and ask them to be alert to their method. If the concerns are major then the Research Manager should do the next interview to demonstrate how the interview should be conducted.
- TREC Central is considering hiring professional actors to pose as health care aides. This approach is used consistently in medicine to teach medical students about how to work with ‘real’ people and in the case of TREC, could be used as a ‘secret shopper’ type of approach. They would pose as aides and after the interview, in a separate location, would complete the form noted above as well as the **Interviewer Checklist (Internal form 1)** and provide these to the respective Research Manager. This information would be used to improve process and highlight areas impacting quality control. The interviewer would not know they were interviewing an actor ahead of time but would be informed as part of the review.

## Yearly

- Time will be dedicated at the TREC annual meeting for practice
  - Interviewers must practice within and between provinces
- TREC Central will monitor the quality of its provincial teams by visiting the various sites. The aims of these visits are:
  - To assess the overall performance of the provincial team
  - To assess the quality of the data collection for current protocols
  - To provide consultation in identifying and solving problems
  - To transfer effective approaches from one province to another
- The site visit team will include the PI and/or one of the co-investigators and/or the Administrative Director.
- TREC Central will update the protocol at least yearly, and more frequently if issues, concerns or new developments arise.

## Assessing the Quality of the Data

### (As it relates to Interviewer Quality Control)

The outcome for the previously described processes is to insure that the interviewer is collecting data of good quality and that the software program and equipment are functioning as intended.

During the data collection period, TREC Central will assess the reported data on an ongoing basis. This is important because these assessments will inform us as to whether the processes are being adequately implemented. The Data Committee and the data unit, including the Data Manager, will be responsible for determining, performing and evaluating the assessments. We have determined that the following quality indicators will serve as outcomes measures for evaluating the quality of data:

- Response rates
- Refusal rates
  - No shows
  - Interrupted interviews
  - Referral to EAP
- Missing data rates
- Typical distribution of responses
- Data entry

### Prior to Data Collection

Each Research Manager, prior to data collection, will provide the Administrative Director with an estimate of the number of staff, by facility, unit (if possible) and job category, that meet (and do not meet) our eligibility criteria. This will be provided to the Data Manager for estimating initial response rates.

### Daily

Interviewers will select forms on a daily basis.

The software company will provide authorized TREC Central staff with live, standardized reports that indicate the number of surveys completed by province, site, unit, and job category. These reports will be compiled from raw, uncleaned data and will be sufficient for initial indications of response rates particularly by regulated staff who complete the survey on their own.

The software company will also provide authorized TREC Central staff with access to the database itself, enabling them to download individual files, as desired, for random checks.

### Weekly

**By the end of Thursday** each week, individual interviewers will submit completed form(s) to their Research Manager who will then collate all forms from all interviewers and submit to the Data Manager (or designate) on Friday morning.

Research Managers will courier (to the Data Manager) all health care aide interviews using a paper version of the survey that have been completed that week. Each survey will be placed in a separate, sealed envelope and indicate the unique identifier assigned to this respondent, on the top right corner. Completed paper interviews have likely happened due to software or equipment malfunction but the responses have since been entered by the interviewer and uploaded to the main database.

The Data Manager (or designate) will:

- Verify the **response rate** **of health care aides** by calculating the number of submitted/completed interviews in the main database and comparing these numbers with the reports from the Research Managers regarding how many interviews have been performed and uploaded in their province. As each upload has one confirmation number assigned to all surveys uploaded at one time, this can serve as another source of confirmation. Any discrepancies will be investigated and immediate actions will be taken to rectify the discrepancy as this could indicate technical problems or lack of skills for uploading the collected data by interviewers
- Calculate HCA **refusals**:
  - Number of eligible HCAs that did not schedule an interview time
  - Number of “no shows” (that is did not come for their scheduled interview)
- Calculate the number of incomplete health care aide surveys (which could indicated interrupted interviews that are pending re-scheduling), by facility
- Calculate the **response rate of regulated staff** by reviewing the standardized reports on number of submitted/completed responses and comparing to the list of eligible staff provided by the Research Managers
- Provide summary reports to the research managers (copy to the Administrative Director) by the end of Friday.

### Monthly

The Data Manager (or designate) will:

- Calculate the number of times the survey was stopped because respondent did not meet criteria (by site and job category)

### Quarterly

On the last day of each quarter (October 31, January 31, April 30 and July 31), the software provider will lock data entry; no further responses will be allowed. Any paused surveys will be considered incomplete.

The software provider will a report on the number of technical calls.

The Data Manager will compile and report on:

- Time to conduct consents, by facility, interviewer and overall
- Overall staff time required to conduct interviews, per facility, using reports provided by Research Managers, including:
  - Travel time
  - Time to complete consent
  - Time to complete survey
  - Time to complete survey checklist
  - Total time doing interviews
  - Average time/interview (completed/incomplete)
- Number of surveys done using paper, by facility and all facilities, combined.

The Data Committee:

- The summary of the responses to the survey’s checklist for surveys completed in each site will be provided to the respective Research Managers for discussion locally with the intent of making any required changes or adjustments in approach.

The Data Committee is responsible for recommending actions to RMC and/or the PI if any atypical patterns occur.

### Yearly

The quality reports from the Data Committee will be presented and discussed at the TREC annual meeting.

The outcome measures will be revised, as needed.

Table 1: Interviewer Quality Control Summary: Project 1

| **Frequency** | **Who** | **What** | **Source(s)** | **Report To** |
| --- | --- | --- | --- | --- |
| **Prior to data collection** | RM* | # Staff, by facility, unit (if possible) and job category, that meet (and do not meet) our eligibility criteria. | Facility and unit administrators | Admin Director/DM |
| **Daily** | Interviewers | Forms x and x (numbers will be assigned shortly) | Forms cabinet | RM |
| **Weekly (Thursday)** | Interviewers | Submit forms X and X (numbers will be assigned shortly) | Forms cabinet | RM |
| **Weekly**  **(Friday)** | RM | Collate forms x and x, by facility | Form # x (TBD) | DM |
|  | RM | Submit paper versions of HCA interviews that have been completed that week |  | DM |
|  | DM | HCA response rate | Central server  Reports from RM | PI, DMC (chair), Admin director; RMs by Friday pm |
|  | DM | HCA refusal rates | Central server  Reports from RM | PI, DMC (chair), Admin director; RMs by Friday pm |
|  | DM | # Incomplete HCA surveys | Central server | PI, DMC (chair), Admin director; RMs by Friday pm |
|  | DM | Regulated staff response rate | Standardized reports  Reports from RMs | PI, DMC (chair), Admin director; RMs by Friday pm |
| **Monthly** | DM | # Of times survey was stopped as respondent did not meet criteria, by site and job category | Central server | Admin director; RMs |
| **Quarterly** | DM | Overall staff time required to conduct interviews, per facility, including:   - Travel time - Time to complete consent - Time to complete survey - Time to complete survey checklist - Total time doing interviews - Average time/interview (completed/incomplete) | Reports from RMs | PI, DMC (chair), admin director, RMs |
|  | DM | # Of surveys done by paper, by facility and all facilities, combined | Central server (survey checklist question +)  Reports from RMs | PI, DMC (chair), admin director, RMs |
| **Quarterly** | DMC | Survey checklist responses, by facility | Data repository | PI, DMC (chair), admin director, RMs |
| **Yearly** | DMC | Report at TREC annual meeting | DMC | Whole team |
|  | TREC Central | Revise protocol as needed | DMC; authors | Whole team |

***RM = research managers; DM = data manager; PI = Principal Investigator; DMC = data management committee**

**Table 2**: Summary of Data Quality Control Steps: General

| **Frequency** | **Who** | **What** | **Source** | **To Whom** |
| --- | --- | --- | --- | --- |
| **Daily** | Nooro | Live, standardized reports that indicate # of surveys completed by province, site, unit, and job category |  | DMC (chair), PI, data mgr, admin director |
|  | Nooro | Access to the main database |  | DMC (chair), PI, data mgr |
| **Monthly** | DM | Look for missing survey data | Central server | DM |
| **Quarterly** | Nooro | Lock database as of end of quarter | Central server | Data mgr |
|  | Nooro | # Of technical help requests | Log | Data mgr, Admin director |
|  | DM | Download locked database, clean according to protocol and save to repository | Central server | PI, DMC (chair) |
|  | DM | Response distribution across provinces, facilities, and units by job category | Data Repository | PI, DMC (chair), admin director |
|  | DM | Response distribution of individual responses to survey questions by interviewer and overall, by facility | Data Repository | PI, DMC (chair), admin director |
|  | DM | Response distribution of individual questions in the survey checklists, by province, facility, and interviewer | Data Repository | PI, DMC (chair), admin director |
|  | DM | # Of times (once, 2 – 3 times, > 3 times) the survey was accessed (prior to submitting) by job category |  |  |
|  | DM | Random data entry checks comparing paper surveys to actual entries | Central server  Paper survey | PI, DMC (chair), admin director |
|  | DMC | Evaluate weekly, monthly, and quarterly reports | DM |  |
|  | DMC | Summary report | RMC |  |

***RM = research managers; DM = data manager; PI = Principal Investigator; DMC = data management committee**

**Interviewer Checklist**

*Instructions: complete after observing an interview*

*Interviewer name:___________ Reviewer name:____________*

*Date: _________*

| **Acceptable** | | **Physical attributes** |
| --- | --- | --- |
| **Yes** | **No** |  |
|  |  | Open posture |
|  |  | Regular eye contact with interviewee |
|  |  | Comfort with response card format |
|  |  | **Personal Characteristics** |
|  |  | Personable, engaging, professional |
|  |  | Appropriate dress |
|  |  | Good hygiene |
|  |  | Appropriate speed of talking; no stumbling or pauses |
|  |  | Clear and audible |
|  |  | Confident with problem-solving during interviews |
|  |  | **Technical Skills** |
|  |  | Able to log on computer knowing IDs/passwords for that machine |
|  |  | Able to open and launch the virtual server software developed specifically for this project |
|  |  | Able to read and accurately enter the unique identifier |
|  |  | Comfortable with using a mouse |
|  |  | Acceptable typing speed |
|  |  | Able to conduct the interview while entering responses with minimal delays |
|  |  | Understands the software sufficiently that there are no surprises as data is entered (e.g., “I wonder why that icon just showed up?”) |
|  |  | Able to open the Internet and connect to the live server to allow data synchronization |
|  |  | **Compliance with TREC policies for:** |
|  |  | Obtaining consent |
|  |  | Conducting interviews |
|  |  | Completion of required tracking forms |
|  |  | Uploading data |
|  |  | Security and safe handling/storage of equipment/documents (e.g., laptops) |
| **General Comments** | | |

Form Int1.

**TREC Survey Interviewer Monitor Form**

*Instructions: The Research Manager will observe interviewers as they actually conduct surveys and indicate adherence to protocol and areas for improvement giving specific examples. A “fault” is when the interviewer deviates from the structured interview format (e.g. provides prompts other than those on screen; adlibs, etc.)*

Interviewer Name: _____________________________ Reviewer name: ________________________ Date: _____________________

|  | **Correct** | **1 Fault** | **2 Faults (or more)** | **Page #** | **Monitor Comments** |
| --- | --- | --- | --- | --- | --- |
| **Interviewer Techniques** |  |  |  |  |  |
| Reads question verbatim and repeats when required. |  |  |  |  |  |
| Demonstrates proper use of response cards. |  |  |  |  |  |
| Provides standardized prompts, when asked for alternate wording. |  |  |  |  |  |
| Appropriate use of secondary scales (e.g. those where ‘never’ means 10% or less of the time and “almost always” means almost 100% of the time). Appropriate=not reading out loud to interviewee. |  |  |  |  |  |
| **Interviewer Delivery** |  |  |  |  |  |
| Interview Flows (speed, no stumbling, pauses, audible) |  |  |  |  |  |
| Conducts interview with enthusiasm and confidence (engaging, friendly, pleasant) |  |  |  |  |  |
| The interviewer is professional (no personal information, no comments to responses) |  |  |  |  |  |
| **Data Entry** |  |  |  |  |  |
| Correctly inputs closed responses (e.g. scales, yes/no responses, picks lists) |  |  |  |  |  |
| Accurately codes ‘other’ responses |  |  |  |  |  |
| Type in relevant information for open-ended responses. |  |  |  |  |  |

Form Int2.0

**EXAMPLE of COMPLETED FORM**

**TREC Survey Interviewer Monitor Form**

*Instructions: The Research Manager will observe interviewers as they actually conduct surveys and indicate adherence to protocol and areas for improvement giving specific examples. A “fault” is when the interviewer deviates from the structured interview format (e.g. provides prompts other than those on screen; adlibs, etc.)*

Interviewers Name: John Smith Reviewer name: Jane Doe Date: 2008/0602

|  | **Correct** | **1 Fault** | **2 Faults (or more)** | **Page #** | **Monitor Comments**  **(Examples of how to provide feedback)** |
| --- | --- | --- | --- | --- | --- |
| **Interviewer Techniques** |  |  |  |  |  |
| Reads question verbatim and repeats when required. | x |  |  |  | She said moderate to severe for her pain. You did well to ask which word works for her. Well done.  Very accurate persistence with scales. This respondent needed a clarification that you did professionally. |
| Demonstrates proper use of response cards. |  | x |  |  |
| Provides standardized prompts, when asked for alternate wording. | x |  |  |  |
| Appropriate use of secondary scales (e.g. those where ‘never’ means 10% or less of the time and “almost always” means almost 100% of the time). Appropriate=not reading out loud to interviewee. | x |  |  |  |
| **Interviewer Delivery** |  |  |  |  |  |
| Interview Flows (speed, no stumbling, pauses, audible) | x |  |  |  | Good pacing, clear neutral voice. You engaged the respondent well and kept the interview flowing., considering there was noise of other staff in the background.  Very good control. He needed to remain focused and you did this very well. |
| Conducts interview with enthusiasm and confidence  (engaging, friendly, pleasant) | x |  |  |  |
| The interviewer is professional  (no personal information, no comments to responses) | x |  |  |  |
| **Data Entry** |  |  |  |  |  |
| Correctly inputs closed responses  (e.g. scales, yes/no responses, picks lists) |  |  | x |  | You seemed to have trouble clicking on the radio buttons.  Professional, enthusiastic and accurate survey. Good job. |
| Accurately codes ‘other’ responses |  |  |  |  |
| Type in relevant information for open-ended responses. |  |  |  |  |

**REFERENCES**

American Statistical Association. (2003). *Interviewer falsification in survey research: Current best methods for prevention, detection, and repair of its effects.* Retrieved June 03, 2008, from <http://www.amstat.org/sections/srms/falsification.pdf>

Balon, R. (2005). By whom and how is the quality of research data collection assured and checked? *Psychotherapy and Psychosomatics, 74*(6), 331-335.

Kobak, K. A., Engelhardt, N., & Lipsitz, J. D. (2006). Enriched rater training using internet based technologies: A comparison to traditional rater training in a multi-site depression trial. *Journal of Psychiatric Research, 40*(3), 192-199.

Loosveldt, G., Carton, A., & Billiet, J. (2004). Assessment of survey data quality: A pragmatic approach focused on interviewer tasks. *International Journal of Market Research, 46*, 65-82.

Martin, R. J., Kephart, D. K., Dyer, A. M., Fahy, J., Kraft, M. (2001). Quality control within the asthma clinical research network. *Controlled Clinical Trials, 22*(6 Suppl), 207S-21S.

Tallmer, J., Scherwitz, L., Chesney, M., Hecker, M., Hunkeler, E., Serwitz, J., et al. (1990). Selection, training, and quality control of type A interviewers in a prospective study of young adults. *Journal of Behavioral Medicine, 13*(5), 449-466
