# Supplementary material for: A Data Quality Control Program for Computer-Assisted Personal Interviews
Source: Nurs Res Pract. 2012 Dec 10;2012:303816. doi: 10.1155/2012/303816 (PMC3529418; doi:10.1155/2012/303816)
Supplement: Supplementary file 2 [file 303816.f2.doc]

# Supplementary File 2

# Data Cleaning and Processing Protocol

**TREC Project 1 (Healthcare Aide Version)**

# Prepared by:

# The *Translating Research in Elder Care (TREC)* Program

## *All cleaning reports are confidential and are to be used internally by TREC data unit. They are not to be used for any analytical purposes as they may not represent the final data sets*

## Objective 1: Data Flow Structure

RAW

DATA

(From Nooro)

EDIT

DATA

(SPSS)

MASTER

DATA

(Cleaned SPSS)

INDEX

DATA

(De-identified with Derived Scales)

## Individual analytical files are derived from the index file

## Objective 2: Systematic Data Entry

1. CAPI survey: Data unit will receive TREC online survey data in ASCII form from Nooro pulled by TREC data manager. The pulled data is RAW DATA.
2. Paper-based HCA survey: Two interviewers will convert paper-based HCA surveys to online surveys; one pretends to be an interviewee to answer survey questions according to the paper-based survey and another one will enter the answers into online survey and upload it to the data base of Nooro.

## Objective 3: Data cleaning [Report A]

***NOTE: Data is considered clean when**:

- **Quarterly**: When Reports A through C are approved and signed off by TREC Project 1 lead investigators. When quarterly data is declared clean the date this occurs must be clearly documented within cleaning report C.
- **Annual**: When Reports A through D are approved and signed off by TREC Project 1 lead investigators. When annual data is declared clean the date this occurs must be clearly documented within cleaning report D.

1. Data analyst writes syntax to convert flat ASCII data file into SPSS data file according to the appropriate TREC survey codebook. To make sure the converting is correct; a data unit junior analyst or trainee will double check the syntax and run the syntax separately. The converted SPSS data file is EDIT DATA.
2. ***Special Cases***: if, for any reason, there is an extension of a quarter and surveys have to be completed using pen and paper, which cannot then be entered into the Nooro system directly, these surveys will be returned to TREC Central. Upon receipt of the surveys, they will be manually coded by a data unit member according to the appropriate version of the TREC survey codebook. Following coding, they will be manually entered by a member of the data unit into the SPSS EDIT DATA file. Two members of the data unit will enter the paper-based HCA surveys: one will read the answers aloud on the survey while another individual enters the answers into the SPSS EDIT DATA file.
3. TREC CAPI data will be cleaned quarterly by a data analyst and a data unit junior analyst or trainee. All results and decisions of each step will be recorded (in tracking tables and in a quarterly cleaning report).
4. Data analyst (and a data unit junior analyst or trainee) will document all errors identified in the cleaning process in a tracking table (see Table 1 at end of this protocol). Corrections to systematic errors will be made where it is logical to do so. Correction of random errors will be made ONLY under the direction of TREC Project 1 lead investigators.
5. Data analyst (and a data unit junior analyst or trainee) will use syntax to check the skip patterns to identify any existing systematic errors and to ensure there is no mixture of “not applicable” and “missing” responses. All skip pattern errors will be recoded in a tracking table (See Table 2 at end of this protocol).
6. Data analyst (and a data unit junior analyst or trainee) will check for ‘out of range’ and ‘wildcard’ values by running frequencies on all variables. All ‘out of range’ or ‘wildcard’ values found will be documented as data errors in Table 1.
7. Data analyst (and a data unit junior analyst or trainee) will check for consistency in variables. For example, VAR002 “Quarter” and VAR010 “submit date” should match. All consistency errors will be recorded in Table 1.
8. Data analyst (and a data unit junior analyst or trainee) will use syntax to do missing pattern checking to ensure the following minimum conditions are met:
   1. maximum percentage of system/or user defined missing value in a given variable=70%
   2. maximum percentage of cases in a single category =95%
   3. maximum percentage of categories with a count of 1=90%
   4. check for variables with no variations
   5. flag empty cases: i.e., cases with all relevant variables are missing or blank

All errors will be recorded in Table 1.

1. Data analyst (and a data unit junior analyst or trainee) will obtain a random sample of paper surveys (25% of sample of paper-based surveys) from the TREC Data Manager and manually check data entry for all variables in these surveys. If the error rate is > 5%, a second random sample will be obtained and the process repeated till the error rate is < 5%. All errors will be recorded in Table 1.
2. The data analyst (and a data unit junior analyst or trainee) will repeat step 6 (frequencies on all variables) (to ensure no new error was introduced when correcting systematic errors). **If no new errors are found, the data will be declared cleaned and a cleaning report (Part A) generated for TREC Project 1 lead investigators.**
3. The data analyst will ***save the cleaned data as MASTER DATA after the TREC Project 1 lead investigators review the cleaning report*** ***(Part A) and provide approval to declare the data as “Master Data”.***

## Objective 4: Pre-derivation [Report B]

1. Data analyst will ensure non-respondents (missing and non applicable) cases for each variable are properly coded as not applicable (8, 88, 8888) and missing (9, 99, 9999) according the appropriate TREC HCA codebook.
2. A combined score for Time on Unit and Time as HCA will be computed.
   1. Total Time as HCA - combining years as HCA with months as HCA to get total time (in years) as HCA
   2. Total Time on Unit - combining years on unit with months on unit to get total time (in years) on unit

***These 2 combined variables will have frequencies and graphs run in report B along with the other survey variables. This is necessary to determine whether all individuals responding meet inclusion criteria.***

1. Data analyst will obtain frequency tables and graphs (histograms, bar charts, etc as appropriate) for all survey variables.
2. Boxplots (as appropriate) for variables in the data set to determine outliers will be run. Recommendations on how outliers should be treated in the analyses to minimize potential effect on the results should be provided to TREC Project 1 lead investigators.
3. Data analyst will obtain distributions of each variable (minimum and maximum values, mean, standard deviation, skewness - normally distributed or skewed, what direction: right, left). Histograms with normal curves will be used in this step.
4. Data analyst will obtain a frequency list for ‘missing’, ‘not applicable’, and ‘not available’ responses. If > 10% of the sample is missing, further exploration should be conducted (e.g., cross tabs with demographic variables). **Suggestions for treating missing values (i.e., list-wise deletion, case-wise deletion, or imputation) are to be provided to TREC Project 1 lead investigators prior to proceeding to deriving variables**. If the data analyst suggests imputations, a report outlining the recommended approaches for missing, invalid or inconsistent data will be prepared (where necessary and appropriate). Final decision on the method for handling missing data will be made by **TREC** **Project 1 lead investigators**.

**Part B of the cleaning and processing report will be generated detailing findings from Objective 4 (pre-derivation) outlined above. This report will be sent to TREC Project 1 lead investigators, who will advise the data analyst if additional investigation is necessary.**

1. Prior to deriving scores for scales within the TREC survey:

- Reverse code each categorical response variable with negative wording
- If required for derived scores, weights will be derived for each respondent. If the data analyst suggests a weighting strategy be employed for select concepts, a report is to be prepared justifying why this strategy is recommended. The final decision as to whether to use a weighting strategy will be made by TREC Project 1 lead investigators.

## Objective 5: Deriving Scales

**The Analyst will check the TREC HCA scoring sheet each quarter prior to deriving scores. *This sheet will be signed and dated each quarter by TREC Project 1 lead investigators.***

1. Using the TREC HCA scoring sheet, the data analyst will derive a score (and name the variable as specified in the scoring sheet) for each of the following concepts in the TREC survey.

- Leadership
- Culture
- Evaluation
- Formal interactions
- Informal interactions
- Social capital
- Structural and electronic resources
- Organizational slack – staffing
- Organizational slack – space
- Organizational slack – time
- CRU
- Attitude towards research
- Belief suspension (implement)
- Aggression
- MBI exhaustion
- MBI cynicism
- MBI efficacy
- Health status pcs8
- Health status mcs8

***Derived scores should also be created for single-item research use variables (dependent variables): IRU, PRU, ORU***

1. **All Individual items will be retained in the data file in addition to the derived scores (as it is anticipated that in some of the analyses, these scores may be used).**

## Objective 6: Descriptive Analysis of Derived Scores [Report C]

**NOTE: Urban and Rural facilities are to be split and looked at separately; two separate report C’s are to be prepared.**

1. Data analyst to obtain frequency tables and boxplots (or histograms as the data dictates) for all derived scores in the data set to determine outliers. Recommendations on how these outliers should be treated in the analyses to minimize potential effect on the results should be provided to **TREC** **Project 1 lead investigators**.
2. Data analyst to obtain distributions for each of the derived scores (minimum and maximum values- theoretical and actual, mean, standard deviation, skewness - normally distributed or skewed, what direction: right, left). This information is to be presented in tabular form.

**Part C of the cleaning and processing report will be generated detailing findings from Objective 6 (descriptive analysis of derived scores) outlined above. This report will be sent to TREC Project 1 lead investigators, who will advise the data analyst if additional investigation is necessary.**

## Objective 7: Assessment of Missing Data [Report D]

**NOTE: Urban and Rural facilities are to be split and looked at separately; two separate report D’s are to be prepared.**

1. Data analyst to obtain frequencies of ‘missing’ responses for each item (including demographics) and derived scores by unit (within each facility) for all three provinces. This data is to be presented in tabular form (See table 3 at the end of this protocol).
2. Data analyst to provide a graph (bar/pareto chart) indicating total number of items respondents missed. For example, how many respondents missed 1 items, missed 2 items, etc.
3. Data analyst to provide a graph (bar/pareto chart) for each derived scale that indicates total number of respondents that missed an item in that scale where a derived score for the scale could not be computed for them. Single items on research use (although not scales) are also to be analyzed in this way.
4. Any items or scales that have **greater then 10% missing data** should be further examined according to:

- respondent age
- shift most often worked
- owner-operator model
- facility size (number of beds)
- English as first language
- who the leader is (var051)
- leadership (derived score), culture (derived score), and evaluation (derived score).

These findings should be presented in tabular or graphical form and Project 1 lead investigators notified of their existence.

1. Suggestions for treating missing values (i.e., list-wise or case-wise deletion or imputation) should be provided by the data analyst. Final decision on how to handle missing data will be made by TREC Project 1 lead investigators.
2. If TREC Project 1 lead investigators determine cases need to be deleted this must be done using syntax. Prior to running the syntax it must be checked by a member of the data unit.

If cases are deleted, scores will need to be re-derived and Report C must be re-generated.

1. **Part D of the cleaning and processing report will be generated detailing findings from Objective 7 (assessment of missing data) outlined above. This report will be sent to TREC Project 1 lead investigators, who will advise the data analyst if additional investigation is necessary.**
2. The data analyst will ***save the*** de-identified data with all derived variables **as INDEX DATA  *after TREC Project 1 lead investigators review the cleaning report*** ***(Parts B & C & D) and provide approval to declare the data as “Index Data”.***

Table 1: TREC HCA Data Cleaning Errors (Wave 1, Quarter 1)

| **Date and initials** | **SUBJECT ID** | **Variable number** | **variable description** | **Error Type (Random or Systematic)** | **OLD ENTRY** | **Should be** | **Action Taken** |
| --- | --- | --- | --- | --- | --- | --- | --- |
| Jan 21, 2009  JS/LLK | 05465 | 128 | Support for continuing education | Systematic | 9 | 8 | fixed by syntax (LLK) |

Table 2: TREC HCA Data Skip Pattern Errors (Wave 1, Quarter 1)

| **Date and initials** | **Variable number and description** | **Error description** | **Action Taken** |
| --- | --- | --- | --- |
| Jan 14, 2009  JS/LLK | VAR027 Worked in HC before coming to Canada  VAR028 Position before coming to Canada | 8 (NA) in VAR027 should be coded as 8 (NA) in VAR028 instead of 9 (Missing) | Wrote syntax to fix it and informed Shane to correct it in his coding |

Table 3: Missing Data Assessment (Wave 1) by Unit and Nursing Home

| ITEM | **NH24** | | | | **NH25** | | | | | | **NH26** | | | | **NH27** | | | | **NH28** | | | **NH29** | | | | | **NH30** | | | | | |
| --- | --- | --- | --- | --- | --- | --- | --- | --- | --- | --- | --- | --- | --- | --- | --- | --- | --- | --- | --- | --- | --- | --- | --- | --- | --- | --- | --- | --- | --- | --- | --- | --- |
|  | U1 | U2 | U3 | Sum | U1 | U2 | U3 | U4 | U5 | Sum | U1 | U2 | U3 | Sum | U1 | U2 | U3 | T | U1 | U2 | Sum | U1 | U2 | U3 | U4 | Sum | U1 | U2 | U3 | U4 | U5 | Sum |
| N (%) missing | | | | | | | | | | | | | | | | | | | | | | | | | | | | | | | | |
| L1… |  |  |  |  |  |  |  |  |  |  |  |  |  |  |  |  |  |  |  |  |  |  |  |  |  |  |  |  |  |  |  |  |
| L2… |  |  |  |  |  |  |  |  |  |  |  |  |  |  |  |  |  |  |  |  |  |  |  |  |  |  |  |  |  |  |  |  |
| L3… |  |  |  |  |  |  |  |  |  |  |  |  |  |  |  |  |  |  |  |  |  |  |  |  |  |  |  |  |  |  |  |  |
| L4… |  |  |  |  |  |  |  |  |  |  |  |  |  |  |  |  |  |  |  |  |  |  |  |  |  |  |  |  |  |  |  |  |
| L5… |  |  |  |  |  |  |  |  |  |  |  |  |  |  |  |  |  |  |  |  |  |  |  |  |  |  |  |  |  |  |  |  |
| L6… |  |  |  |  |  |  |  |  |  |  |  |  |  |  |  |  |  |  |  |  |  |  |  |  |  |  |  |  |  |  |  |  |
| Leadership (derived) |  |  |  |  |  |  |  |  |  |  |  |  |  |  |  |  |  |  |  |  |  |  |  |  |  |  |  |  |  |  |  |  |
| Etc. |  |  |  |  |  |  |  |  |  |  |  |  |  |  |  |  |  |  |  |  |  |  |  |  |  |  |  |  |  |  |  |  |
|  |  |  |  |  |  |  |  |  |  |  |  |  |  |  |  |  |  |  |  |  |  |  |  |  |  |  |  |  |  |  |  |  |
|  |  |  |  |  |  |  |  |  |  |  |  |  |  |  |  |  |  |  |  |  |  |  |  |  |  |  |  |  |  |  |  |  |
